# Development and optimization of an ammonia removal strategy for sustainable recycling of cell culture spent media in cultivated meat production: from concept to implementation

**DOI:** 10.3389/fbioe.2025.1617115

**Published:** 2025-08-06

**Authors:** Babak Pakbin, Armaghan Amanipour, Arian Amirvaresi, Arash Shahsavari, Reza Ovissipour

**Affiliations:** Department of Food Science and Technology, Texas A&M University, College Station, TX, United States

**Keywords:** cultivated meat, spent media, recirculating media, optimization, response surface methodology, lamb muscle cell growth

## Abstract

**Introduction:**

Ammonia is a toxic metabolic waste produced during mammalian cell metabolism, with inhibitory effects against cell growth.

**Methods:**

This study focuses on developing and optimizing an ammonia removal approach to support spent media recycling throughout sustainable cultivated meat biomanufacturing.

**Results:**

Among the various methods evaluated, the alkalization-stripping method was found to be significantly more efficient and rapid than other strategies to remove ammonia ions while preserving the remaining glucose contents. The optimized process parameters were determined to be a pH of 12 following a 15-minute stripping process, achieving more than 82% ammonia removal efficiency. When applied to lamb satellite cells, the treated spent media improved the cell growth rate without inducing any morphological changes.

**Discussion:**

A 50:50 ratio formulation of treated spent media to fresh media demonstrated an efficient, cost-effective, and environmentally friendly solution for spent media recycling, providing a practical approach to implementing sustainable media recycling in cultivated meat production.

## 1 Introduction

Cell culture techniques have been developed and advanced as a novel approach with various research and industrial applications in medicine and agriculture, such as cancer research, vaccine and hormone production, drug and antibody development, and, more recently, the development of cellular agriculture. Cellular agricultural bioprocesses have been proposed as sustainable strategies to address the current and future challenges of food security and global climate change (Wikandari et al., 2021). Emerging major problems related to the growing population and the harmful environmental impacts of conventional agriculture are driving the demand for sustainable alternatives through cellular agriculture (Chodkowska et al., 2022). Therefore, cell cultivation technologies have been successfully developed and are emerging as novel and promising biotechnological approaches for biomanufacturing cultivated meats, thus enabling production in bioreactors much more efficiently and rapidly than in traditional animal farming. Cultivated meat production and consumption have already been legalized in some countries around the world, such as the United States, Singapore, and Israel, and it is expected to be authorized and approved as a food source in other major countries soon (Santos et al., 2023; Rasmussen et al., 2024). On the other hand, the number of companies focused on scaling up and commercializing cultivated meat products has increased globally in recent years. More than 100 companies around the world, located in North America, Europe, and Asia, are now focusing on biomanufacturing cultivated beef, seafood, poultry, pork, and some exotic meats such as kangaroo and horse. However, despite the blossoming development of cultivated meat research and industry, numerous challenges remain to be addressed (Kirsch et al., 2023; Lee et al., 2024).

Economically and technically, the development, reuse, and recycling of cell culture media have consistently been among the most serious and significant challenges in cell culture techniques and cellular agriculture. It has been estimated that 55%–95% of the total cultivated meat production cost is attributed to providing the culture medium (Yang et al., 2023). Despite their cost and scalability limitations, commercially available cell culture media, such as Dulbecco’s modified Eagle medium (DMEM) and Roswell Park Memorial Institute (RPMI) cell culture media, are widely used in cell line development and *in vitro* assays for cell biology research (Myers et al., 2023). However, as cellular agriculture and cultivated meat biomanufacturing transition to industrial-scale production, it is imperative to prioritize sustainable, cost-effective, and scalable alternatives (Mattick et al., 2015). These include the development of novel cell culture media formulations and the implementation of cell culture medium recycling strategies (Yang et al., 2023). From the perspective of the first strategy, different plant-based and animal-free cell culture medium formulations have been developed, with ongoing efforts dedicated to creating and optimizing additional formulations (O'Neill et al., 2021; Reiss et al., 2021). Since it has been estimated that as much as 42 L of cell culture media are required to produce 1 kg of cultivated meat, recycling and reusing culture media can be prioritized over the development of new media—particularly considering the critical aspects of sustainability and cost-effectiveness (Yang et al., 2023; Reiss et al., 2021). Through cell metabolic activities, nutrients in the cell culture medium are consumed, and specific waste metabolites are simultaneously produced, which can accumulate and significantly inhibit cell growth and productivity. The removal of these inhibitory metabolites presents a critical challenge to effective recycling and reuse of cell culture media (Yang et al., 2023; O'Neill et al., 2021; Woiciechowski et al., 2024).

Ammonium (NH_4_^+^) is one of the most challenging metabolic waste products generated during cellular metabolism, exerting inhibitory and toxic effects on cell growth. Therefore, the minimization of ammonia concentration is critical for developing a culture media recycling approach that supports achieving high cell densities in cellular agriculture biomanufacturing (Zhang et al., 2022; Hertz et al., 2017). To date, there have been very few efforts to develop and optimize efficient strategies for removing ammonia from spent cell culture media. In addition to ammonium ions, the spent media also contain significant amounts of glucose, lactate, glutamine, and some minerals such as sodium, potassium, and calcium (O’Neill et al., 2022; Elliott et al., 2020). Ammonia was selected as the target in this study due to its more pronounced cytotoxicity than that of lactate under non-replaced medium conditions, although the literature suggests that ammonia accumulation exerts a more immediate inhibitory effect on cell growth during the early phases of culture (Zhang et al., 2022; Saldanha et al., 2023; Yang et al., 2025). An optimal and sustainable approach for ammonia removal from spent culture media should achieve high ammonia removal rates while minimizing the detrimental effects on glucose integrity and other nutrients (Yang et al., 2023; O’Neill et al., 2022). The cost-effectiveness of the developed method should also be carefully considered. A membrane-based system was previously designed and successfully developed to remove toxic ammonia from spent cell culture media; however, limited consideration was given to the cost-effectiveness of the method due to the significantly high cost required to provide the membranes (Martins et al., 2024). Since ammonia is a significant pollutant and a critical water quality parameter, the most effective and affordable ammonia removal approaches were primarily tailored for application in wastewater treatment processes (Kinidi et al., 2018). There are several approaches for removing ammonia ions from wastewater, including ion exchange, membrane filtration, chemical precipitation, ion adsorption, photocatalytic oxidation, and air stripping methods (Ye et al., 2018). We hypothesize that these cost-effective and efficient ammonia removal strategies also have the potential to remove ammonium ions from spent cell culture media while minimizing the detrimental impacts on essential nutrients, mainly glucose, thus providing a sustainable spent media recycling strategy.

To date, there have been a limited number of studies on the development, optimization, or evaluation of an effective and cost-efficient ammonia removal strategy from spent media, leaving a significant gap in providing a practical solution for media recycling in cultivated meat production. Therefore, we first aimed to test our hypothesis by employing and modifying, if necessary, the wastewater ammonia recovery methods to remove ammonium ions from the spent media. We then aimed to model and optimize the process parameters of the selected method using a widely recognized and robust statistical modeling and design of the experiment strategy—the response surface methodology (RSM)—which enabled us to identify the most efficient process parameters. We ultimately sought to assess the proliferation activity of cultured meat cells in recycled spent media treated with the optimized selected approach and subsequently developed and optimized a recycling strategy based on a formulation of treated spent media and fresh media.

## 2 Materials and methods

### 2.1 Spent cell culture medium preparation and collection

Lamb satellite cells (LSCs) used in this study were previously isolated in our laboratory using the method previously described by Stout et al. (2023). LSCs were incubated in DMEM (Thermo Fisher Scientific) supplemented with 20% fetal bovine serum (FBS, Thermo Fisher Scientific), 1% antibiotic–antimycotic solution (Thermo Fisher Scientific), and 1 ng/mL human fibroblast growth factor-2 (FGF-2, Thermo Fisher Scientific) at 37°C with 5% CO_2_ for 72 h at more than 80% confluence. The spent media was harvested by centrifugation at 1,800 rpm for 5 min and was passed through a 0.22-µm filter membrane (Stout et al., 2023). Spent cell culture media from lamb muscle cell cultivation, containing 1.214 ± 0.029 mmol/L ammonium ions (NH_4_^+^), was used as the spent media in this study. After harvesting, the remaining glucose level in the spent media was 2.717 ± 0.053 g/L. The initial levels of ammonia ions and glucose in the regular growth medium (DMEM) were 0.176 ± 0.002 mmol/L and 4.946 ± 0.011 g/L, respectively. The pH value of the collected spent media was also measured to be 7.42 ± 0.08.

### 2.2 Ammonia removal strategies

In this study, we initially evaluated four practical and well-known strategies previously employed for ammonia removal from wastewater—alkalization-stripping, zeolite, struvite precipitation, and titanium dioxide nanoparticles (TiO_2_-NPs)—to remove ammonia ions from cell culture spent media while minimizing any significant impact on glucose content. All the ammonia removal strategies applied to spent media samples in this study were performed under aseptic conditions. To reduce experimental error, all treatments were carried out in biological triplicates, and each experiment was conducted in technical triplicates. The most efficient strategy was then selected for the development and optimization of a targeted ammonia removal strategy from the spent media.

### 2.3 Alkalization-stripping method

The alkalization-stripping method, a modified air-stripping-based ammonia removal strategy involving pH adjustment and high-speed vortexing (1,400 rpm), was used in this study to remove ammonia from spent media. We used diluted 0.1 N NaOH and 0.1 N HCl for pH adjustment. The pH values of the spent media samples were adjusted to pH = 11, and the samples were then subjected to high-speed vortexing at 1,400 rpm at room temperature (RT = 25°C) for 30 min and 120 min. The temperature was maintained at RT during the process (Kinidi et al., 2018). After the treatment, the pH values of the samples were adjusted back to the initial pH value (pH = 7.4), and they were kept at −20°C until further chemical analysis. Untreated spent media (pH = 7.4) were considered the negative controls for all treatments. Fresh standard growth medium was also treated and used as a control in this study.

### 2.4 Zeolite method

In this study, commercial agricultural-grade natural zeolite granules (1.0–1.5 mm granule size) were purchased from the local market and supplier (VORganic, Vermont Organics Reclamation Co., VT, United States) and utilized as the absorbent material to remove ammonia ions from the spent media. To eliminate dust and water-soluble residues, zeolite granules were washed three times with distilled water and then dried using the oven at 105°C overnight. Before their use, zeolite granules were sterilized by autoclaving at 105°C for 20 min. For the ammonia removal treatment of the spent media, zeolite granules were added to the spent media samples at concentrations of 5 g/L and 20 g/L. The samples were then incubated at RT with shaking at 180 rpm for 1 and 4 days. Following the treatments, the zeolite granules were separated by filtration through the sterile 0.45-µm syringe filters, and the treated samples were stored at −20°C until further analysis (Kameda et al., 2021).

### 2.5 Struvite precipitation strategy

The struvite precipitation method was also used as another practical strategy to remove the ammonia ions from the spent media in this study. As previously described by Diwani et al. (2007), magnesium, ammonium, and phosphorus were used in a ratio of 1.6:0.6:1 and at equal molar concentrations based on the initial ammonia concentration in spent media (1.214 ± 0.029 mmol/L). Accordingly, specific amounts of MgCl_2_ (3.237 mmol/L) and KH_2_PO_4_ (2.023 mmol/L) were added to the spent media for struvite formation, and the samples were then shaken at 160 rpm at RT for 60 min. After the treatment, the samples were centrifuged at 4,000 rpm for 20 min for struvite separation and stored at −20°C until chemical analysis (Lorick et al., 2020; El Diwani et al., 2007).

### 2.6 TiO_2_-NP strategy

The TiO_2_-NP strategy was also used as a novel wastewater nitrogen removal method to remove ammonia ions from the spent media in the present study. Commercially available, UV-activated TiO_2_-NPs (Sigma-Aldrich, Darmstadt, Germany) were utilized in this study to treat spent media and remove ammonia ions (Zheng et al., 2011). The primary particle size of the TiO_2_-NPs, as reported by Sigma-Aldrich, was 21 nm. In the present study, 0.05 g/L and 1 g/L concentrations of TiO_2_-NPs were added to the spent media samples, and they were then shaken at 200 rpm at RT for 1 and 5 days. Centrifugation at 3,800 rpm for 15 min was used to remove TiO_2_-NPs from the samples (Hashemi et al., 2022; Baqer et al., 2021). Treated spent media was stored at −20°C until further analysis.

### 2.7 Quantification of ammonium ions and glucose content

Glucose and ammonium ion concentrations in the spent media and control samples were measured using the BioProfile FLEX2 Automated Cell Culture Analyzer machine and the Chemistry Module (Nova Biomedical, Waltham, MA, United States), according to the manufacturer’s instructions. All measurements were carried out in technical triplicate (De et al., 2010).

### 2.8 Experimental design and analysis

Based on the results obtained from the initial evaluation of different ammonia removal strategies to remove ammonia and protect glucose in spent media, we selected the alkalization-stripping strategy for development and optimization. In this study, response surface methodology based on central composite design (RSM-CCD) was employed to describe and optimize the processing parameters of the alkalization-stripping method. The pH value and processing time were used as independent variables, while the ammonium concentration reduction—or ammonia removal efficiency (ARE)—was the sole response (*Y1*) or dependent variable (Table 1). ARE was measured using the following formula:

$$\text{ARE }\left(\%\right)=\frac{A0-A}{A0}\times 100.$$

**TABLE 1:** Experimental design of the process dependent and independent variables.

Variable name	Independent/dependent	Symbol	Coded levels (-α, −1, 0, +1, +α)				
			−1.41	−1	0	+1	+1.41
pH value	Independent	X1	5.96	7	9.5	12	13.03
Time (min)	Independent	X2	8.78	15	30	45	51.21
ARE^a^ (%)	Dependent	Y1					

^a^ ARE: ammonia removal efficiency.

Here, *A* and *A0* represent the ammonia concentration after the treatment and the initial ammonia concentration (*A0* = 1.214 mmol/L), respectively. Since the glucose content was not affected significantly during the process, this factor was not treated as a separate response variable.

Based on the initial evaluation results, the experimental conditions for the tests were also determined and are summarized in Table 1. RSM-CCD was implemented using Design-Expert software version 13.0.5.0 (Stat-Ease Inc., MN, United States). The following quadratic equation describes the behavior of the process variables:

$$Y1=A_{0}+\sum_{i=1}^{n}AiXi+\sum_{i=1}^{n}AiiX^{2}i+\sum_{i\neq 1,j=1}^{n}AijXiXj+\varepsilon .$$

Here, *Y1* represents the response; A_0_ represents the fixed response value determined at the central point of the experimental design; *Ai*/*Aj*, *Aii*, and *Aij* denote the linear, quadratic, and second-order interaction coefficients, respectively; *Xi* and *Xj* represent the independent variables; n represents the number of independent variables; and ε represents the random error. As previously described and suggested by Montgomery (2017), the correlation coefficient, F value, adequate precision ratio, and lack of fitness were measured to evaluate the accuracy, significance, signal-to-noise ratio, and fitness of the fitted model, respectively, and improve the model if necessary. Based on the number of factors (k = 2; non-central points, n = 8) and central points (n = 6), 14 experiments were designed and conducted in this study.

### 2.9 Effects of treated media on cell characterization

To evaluate the cell proliferation activity of the treated spent media for recycling and reusing, the treated and reference samples were characterized for cell growth, activity, and viability. These characterizations include short-term growth, live/dead cell viability, and immunostaining analysis of the LSCs.

### 2.10 Culture and maintenance of LSCs

Prior to cell assessment, the cells were cultured in DMEM (Thermo Fisher Scientific) supplemented with 1 ng/mL of FGF-2 (Thermo Fisher Scientific), 20% FBS (Thermo Fisher Scientific), and 1% antibiotic–antimycotic solution (Thermo Fisher Scientific). Before cell seeding, the flasks were coated with 0.1% (W/V) gelatin (VWR). The cells were grown to approximately 80% confluency and then harvested using a 0.25% trypsin–EDTA solution (Thermo Fisher Scientific). The applied environmental conditions for the cells’ incubation and maintenance were 37°C and 5% CO_2_.

### 2.11 Short-term growth study

The growth rate of LSCs was evaluated in a media sample over 3 days. A 96-well plate (VWR) coated with 0.1% w/v gelatin (VWR) was incubated at 37°C for 30 min. The cells were then seeded at 10^3^ cells per well containing the standard medium and incubated overnight at 37°C and 5% CO_2_ to allow adherence. The medium was then removed, and the cells were washed with Dulbecco’s phosphate-buffered saline (DPBS, MilliporeSigma).

After aspirating with DPBS, fresh standard growth medium (defined as the control medium) containing DMEM (Thermo Fisher Scientific) supplemented with 1 ng/mL of FGF-2 (Thermo Fisher Scientific), 20% FBS (Thermo Fisher Scientific), and 1% antibiotic–antimycotic solution (Thermo Fisher Scientific). Spent media (defined as the negative control), treated spent media (defined as the main treatment containing spent media treated according to the optimized process characterizations in this study), and treated fresh standard growth medium were added to the cells. The LSCs were incubated for 72 h at 37°C with 5% CO_2_. After the incubation time, the cultured LSCs were imaged using an inverted microscope (CKX53 Olympus). The CyQUANT™ NF Cell Proliferation Assay Kit (Thermo Fisher Scientific) was used to evaluate cell proliferation according to the manufacturer’s protocols. All experiments were performed in three biological and three technical replicates to ensure reliability and accuracy (Stout et al., 2023; Chen et al., 2023).

### 2.12 Live/dead cell viability assay

Live/dead imaging was carried out on the third day of the short-term study to assess the cell viability in the treated and control media that demonstrated the highest performance in the CyQUANT assay. The LIVE/DEAD Cell Imaging Kit (Thermo Fisher Scientific), containing calcein AM and BOBO-3 iodide to label live cells in green and dead cells in red, respectively, was used to stain LSCs cultured in the fresh standard growth medium, spent media, and treated spent media, following the manufacturer’s protocols. Following a 30-min incubation at room temperature, the LSCs were washed with DPBS and subsequently imaged using a fluorescence microscope (Stout et al., 2023).

### 2.13 Immunostaining analysis

The PAX7 immunostaining assay was employed in this study to assess the cell differentiation potential and proliferation of LSCs cultured in the fresh standard growth medium, spent media, and treated spent media. After fixation with 4% paraformaldehyde (Thermo Fisher Scientific) at room temperature for 30 min, the cells were permeabilized for 20 min with 0.5% Triton X-100 (MilliporeSigma). To prevent nonspecific binding, the LSCs were blocked for 1 h with 5% bovine serum albumin (BSA) (Thermo Fisher Scientific). The primary antibody was incubated at 4°C overnight using anti-PAX7 (1:100 dilution, Thermo Fisher Scientific, #PA5-68506). The LSCs were then washed with PBS and incubated for 1 h at room temperature with the secondary antibody (anti-rabbit 1:500 in blocking buffer, Thermo Fisher Scientific, #A-11072) in the dark. The nuclei were subsequently stained for 15 min with DAPI (Abcam, #ab104139; 1:200 dilution, Abcam). After a single wash with PBS, the cells were imaged using a fluorescence microscope (Stout et al., 2023).

### 2.14 Evaluation of the recycling strategies

To evaluate and develop the recycling strategy, the growth rate of LSCs cultured in different formulations of treated spent media and fresh media was evaluated using the short-term growth assay (Stout et al., 2023). The recycling formulations used in this study consisted of treated spent media mixed with fresh media at ratios of 90:10, 80:20, 70:30, 60:40, and 50:50.

## 3 Results

### 3.1 Evaluation of ammonia removal methods

**FIGURE 1:**
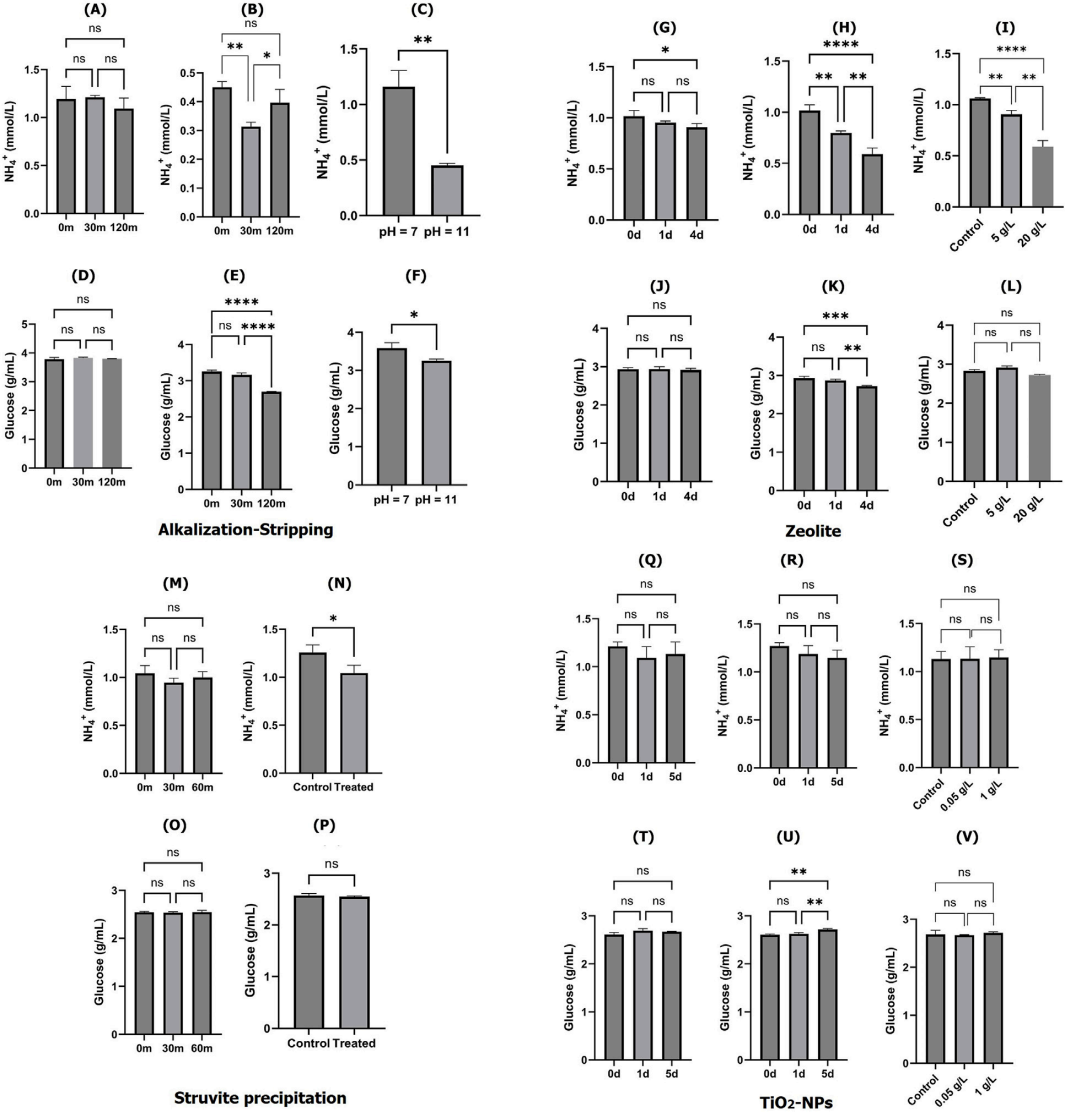
Ammonia and glucose concentrations in cell culture spent media treated with different ammonia removal strategies: ammonia content after alkalization-stripping at pH = 7 **(A)**; ammonia content after alkalization-stripping at pH = 11 **(B)**, ammonia content after 120 min of alkalization-stripping **(C)**; glucose content after alkalization-stripping at pH = 7 **(D)**; glucose content after alkalization-stripping at pH = 11 **(E)**, glucose content after 120 min of alkalization-stripping **(F)**; ammonia content after treatment with zeolite at 5 g/L concentration **(G)**; ammonia content after treatment with zeolite at 20 g/L concentration **(H)**; ammonia content after 4 days of treatment with zeolite **(I)**; glucose content after treatment with zeolite at 5 g/L concentration **(J)**; glucose content after treatment with zeolite at 20 g/L concentration **(K)**; glucose content after 4 days of treatment with zeolite **(L)**; ammonia content after treatment with struvite precipitation approach **(M)**; ammonia content after 60 min of struvite precipitation treatment **(N)**; glucose content after treatment with struvite precipitation approach **(O)**; glucose content after 60 min of struvite precipitation treatment **(P)**; ammonia content after treatment with activated TiO_2_-NPs at 0.05 g/L concentration **(Q)**; ammonia content after treatment with activated TiO_2_-NPs at 1 g/L concentration **(R)**; ammonia content after 5 days of treatment with activated TiO_2_-NPs **(S)**; glucose content after treatment with activated TiO_2_-NPs at 0.05 g/L concentration **(T)**; glucose content after treatment with activated TiO_2_-NPs at 1 g/L concentration **(U)**; and glucose content after 5 days of treatment with activated TiO_2_-NPs **(V)**. *, **, ***, and **** indicate different significance levels. Statistical significance levels were evaluated using one-way ANOVA with Duncan’s multiple range test. Comparisons are between all samples.

The initial goal of this study was to find an appropriate strategy for removing ammonia ions and preserve the remaining glucose in the spent media; therefore, we aimed to develop wastewater treatment-based ammonia removal methods since these approaches are significantly more affordable and efficient. To treat the spent media, four cost-effective and efficient ammonia removal strategies were selected for this study, namely, alkalization-stripping, zeolite, struvite precipitation, and titanium dioxide nanoparticles. The initial ammonia (NH_4_^+^) and glucose concentrations in cell culture spent media were measured at 1.214 ± 0.029 mmol/L and 2.717 ± 0.053 g/L, respectively, in this study. The significant effects of the alkalization-stripping strategy on the ammonia and glucose levels in treated spent media under pH conditions of 7 (neutral) and 11 (alkaline) after 30 min and 120 min are illustrated in Figures 1A–F. No significant decrease was observed in ammonia concentration after 30 min and 120 min of stripping treatment at the neutral pH (pH = 7; Figure 1A); however, the ammonia concentration was significantly (*p* < 0.005) decreased after 30 min of stripping treatment at the alkaline pH (pH = 11; Figure 1B). An alkalization treatment (pH value changing from 7 to 11) contributed to a significantly (*p* < 0.05) lower ammonia concentration in the stripped spent media (Figure 1C). There was no significant change in the glucose content during the stripping process at the natural pH (Figure 1D); however, the glucose content was significantly (*p < 0.0001*) affected and decreased by the treatment at the alkaline pH level (Figure 1E). The effect of alkalization on the glucose content in stripped spent media was significant (*p* < 0.05), but the change was relatively minor (Figure 1F).

The effects of using zeolite as an ion exchange strategy to remove ammonia and preserve glucose in the spent media using different zeolite concentrations (5 and 20 g/L) during 4 days of treatment are illustrated in Figures 1G–L. The ammonia ions and glucose concentrations were relatively stable during the process at a 5 g/L concentration of zeolite (Figures 1G,J). In a time-dependent manner, ammonia ions were significantly decreased after 1 (*p* < 0.005) and 5 days (*p* < 0.0001) of treatment (Figure 1H). As shown in Figure 1I, the zeolite treatment also dose-dependently decreased the content of ammonia ions significantly (*p* < 0.0001); however, no significant zeolite dose-dependent effects were observed on the glucose content. The struvite precipitation strategy was not significantly able to affect the ammonia and glucose contents in the spent media (Figures 1M–P); however, a slight but statistically significant decrease (*p* < 0.05) was observed in ammonia concentration, which was observed, though it was comparatively negligible (Figure 1N). Glucose and ammonia levels were also not significantly decreased in the spent media treated with various concentrations (0.05 and 1 g/L) of TiO_2_-NPs after 5 days of treatment (Figures 1Q–V). To summarize the findings at this stage, the alkalization-stripping and zeolite methods were capable and struvite precipitation and TiO_2_-NP approaches were incapable of removing ammonia and preserving glucose in spent media. Since the alkalization-stripping method proved to be remarkably more efficient and faster than the zeolite strategy, we proceeded with it, ultimately developing an optimized approach for ammonia removal from spent media.

### 3.2 Optimization of the selected method by RSM-CCD

According to the preliminary data obtained from the first part of this study, we proceeded with the process characterizations outlined in Table 1. A total of 14 experimental runs were conducted using a central composite design, with the pH value (A) and time of the treatment (B) as independent variables and ARE% as the single response; the experimental conditions and results are presented in Table 2. We also observed that the glucose content was significantly unaffected by the factors in this study. Consequently, we decided to exclude the glucose content as a dependent variable from the experimental design. The observed percentages of ammonia removal efficiency for the selected method in this study varied between 38.63% and 98.43%.

**TABLE 2:** Experimental design including different factors and response values.

Run no.	Factor	Factor	Response
	A	B	1
	pH	Time	ARE
		Min	%
1	9.5	8.8	72.5
2	12	45	76.6
3	9.5	51	70.6
4	9.5	30	46.1
5	7	15	50.6
6	12	15	81.3
7	9.5	30	54.3
8	5.9	30	53.1
9	9.5	30	49.3
10	9.5	30	38.6
11	7	45	51.9
12	9.5	30	44.4
13	9.5	30	46.4
14	13	30	98.4

The analysis of variance for the fitted quadratic response surface model of ammonia removal from spent media, along with the associated regression parameters, is presented in Table 3. As provided in Table 3, the *F-* (*F-value = 26.20*) and *p-*values (*p* < 0.0001) of the model indicated that the alkalization-stripping process was significantly modeled to remove ammonia from the spent media. *p-values* less than *0.05* showed that the coefficients of the model terms, including the pH value (*p < 0.0001*) and time (*p = 0.7291*), were significant and insignificant, respectively. The second order of all the factors (*A* ^*2*^ and *B* ^*2*^) was also significant. Other coefficients of the model terms were not significant; therefore, they were eliminated to simplify the model. The lack of fitness of the quadratic model was statistically significant (*p* = 2,861). The fit of the model to the experimental data for ammonia removal from spent media was evaluated using the determination coefficients, including *R* ^*2*^ (*R* ^*2*^ = 0.92) and adjusted *R* ^*2*^ (*R* ^*2*^_*adj*_ = 0.88), which were higher than 0.8 (Montgomery, 2017). The adequate precision ratio of the fitted model was measured at 13.24 in this study. The final coded regression quadratic model is presented using the following second-order polynomial equation:

$$\text{ARE }\left(\%\right)=46.53+15.23A\;–\;0.7516B+12.77A^{2}+10.30B^{2}.$$

**TABLE 3:** Analysis of variance for the fitted quadratic model.

Source	Sum of squares	Degree of freedom	Mean square	F-value	*P*-value	Statistical significance
Model	3,709.18	4	927.29	26.20	<0.0001	Significant
A-pH	1856.37	1	1856.37	52.45	<0.0001	
B-Time	4.52	1	4.52	0.1277	0.7291	
A^2^	1,203.80	1	1,203.80	34.01	0.0002	
B^2^	782.91	1	782.91	22.12	0.0011	
Residual	318.54	9	35.39			
Lack of fit	183.38	4	45.84	1.70	0.2861	Not significant
Pure error	135.17	5	27.03			

SD, 5.95; PRESS, 1,060.62; R^2^ = 0.9209; R^2^_adj_ = 0.8858; R^2^_pred_ = 0.7367; Adeq precision = 13.2414.

**FIGURE 2:**
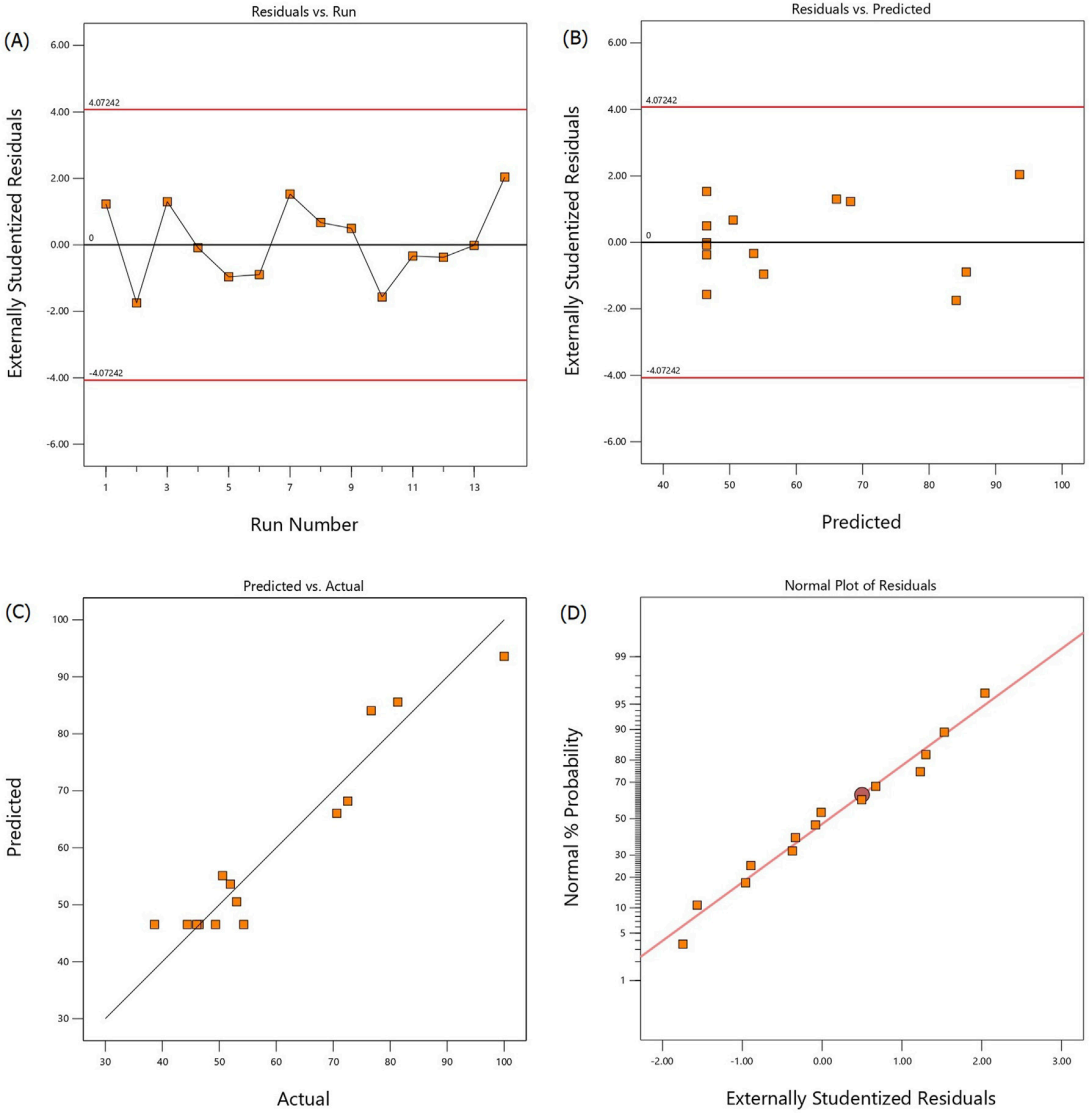
Studentized residuals vs. run number **(A)**, studentized residuals vs. predicted values **(B)**, predicted vs. actual values **(C)**, and normal probability vs. studentized residuals **(D)** plots for ammonia removal efficiency during the alkalization-stripping process modeled and optimized using RSM-CCD to recover ammonia from cell culture spent media.

Model fitting plots of the alkalization-stripping process for ammonia removal from spent media including the studentized residuals vs. runs, studentized residuals vs. predicted values, predicted vs. actual values plots, and the normal probability plot of the studentized residuals are shown in Figures 2A–D, respectively. Figures 2A, B illustrate that most of the experimental running points are randomly distributed and all values range from −4 to 4, indicating that the proposed model has been fitted satisfactorily. The predicted values of the ammonia removal efficiency rates (Figure 2C) obtained from the actual experiments were in correspondence with those from the model. The normal probability of the residuals confirmed the normal distribution of the standard deviations between the actual and predicted responses. The normal probability plot (Figure 2D) shows a normal distribution of the residuals following a straight line, indicating that the fitted model can be used for navigating the design spaces.

**FIGURE 3:**
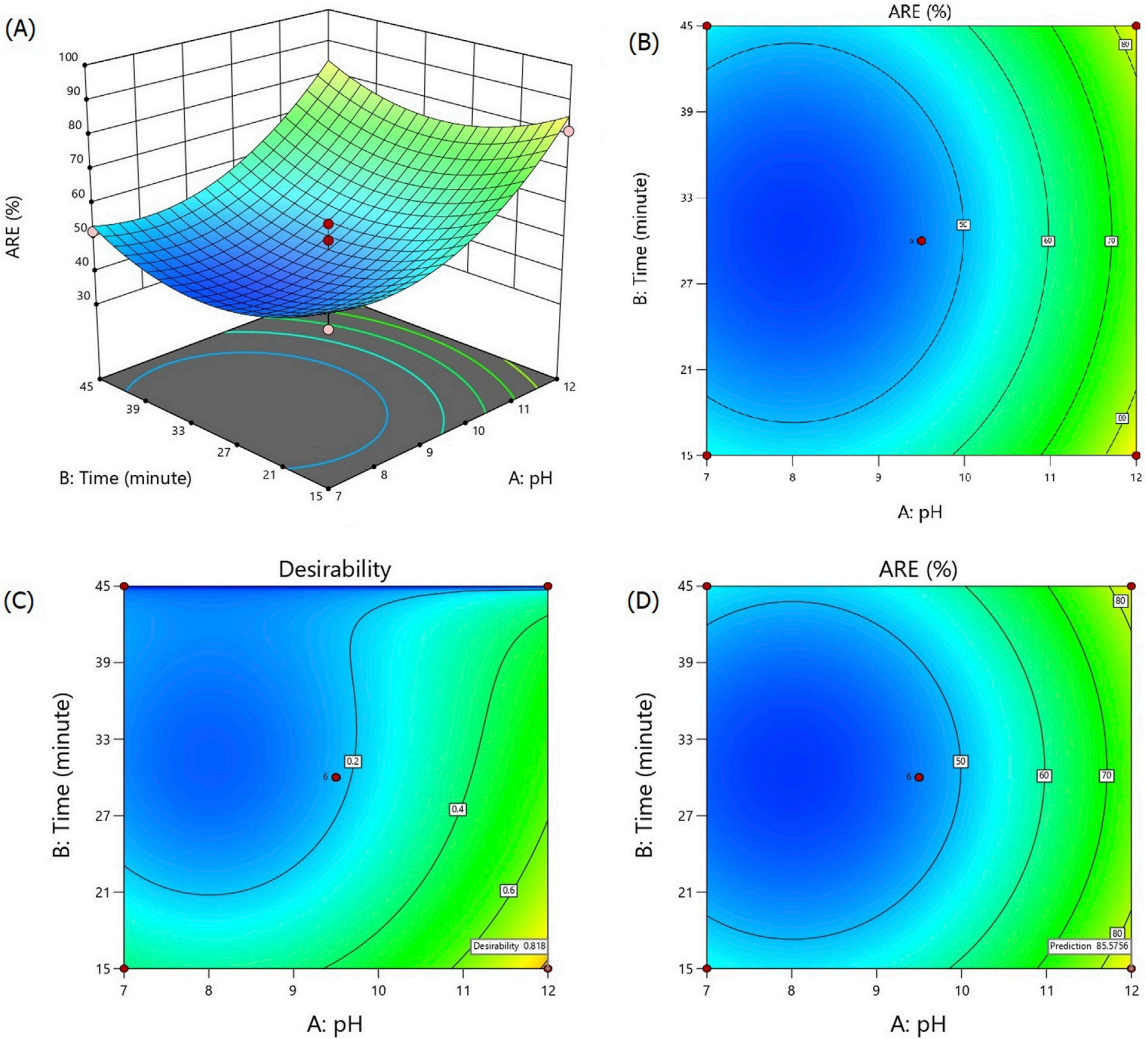
Response surface **(A)**, contour **(B)**, desirability **(C)**, and prediction **(D)** plots for ammonia removal efficiency during the alkalization-stripping process modeled and optimized using RSM-CCD to recover ammonia from cell culture spent media.

The 3D response surface and contour plots of the fitted quadratic model are illustrated in Figures 3A, B, showing the effects of different pH levels and treatment duration in the alkalization-stripping method on ammonia removal efficiency in spent media. The maximum observed ARE was 98.43% at the pH value of 13.05 after 30 min of alkalization-stripping treatment. The minimum ARE percentages were observed at the central experimental points, including the pH value of 9.5 after 30 min of treatment. As shown in Figures 3A, B, increasing pH values led to an increase in ARE, with a particularly dramatic increase observed after a pH value of 9.5. The response of ARE was very sensitive to the pH changes, and the influence of this factor was significantly greater than that of the process duration on ARE. The process parameters were also optimized by RSM-CCD, reaching the optimum value of ARE. The desired goal for the factors and response including the pH value, time, and ARE% were chosen as “in range,” “minimize,” and “maximize,” respectively. As shown in Figure 3C, D, an ARE of 85.57% with more than 81% desirability was predicted according to the optimized processing conditions, including the pH value of 12 after 15 min of stripping treatment. An additional experiment according to the optimized operational conditions (pH value = 12; time = 15 min) was carried out to confirm the optimization result; ultimately, an ARE of 82.45% (0.213 mmol/L ammonia concentration) was obtained, which was reasonably and significantly (*p* < 0.05) close to the predicted value in this study.

### 3.3 Effects of treated spent media on cell characterizations

**FIGURE 4:**
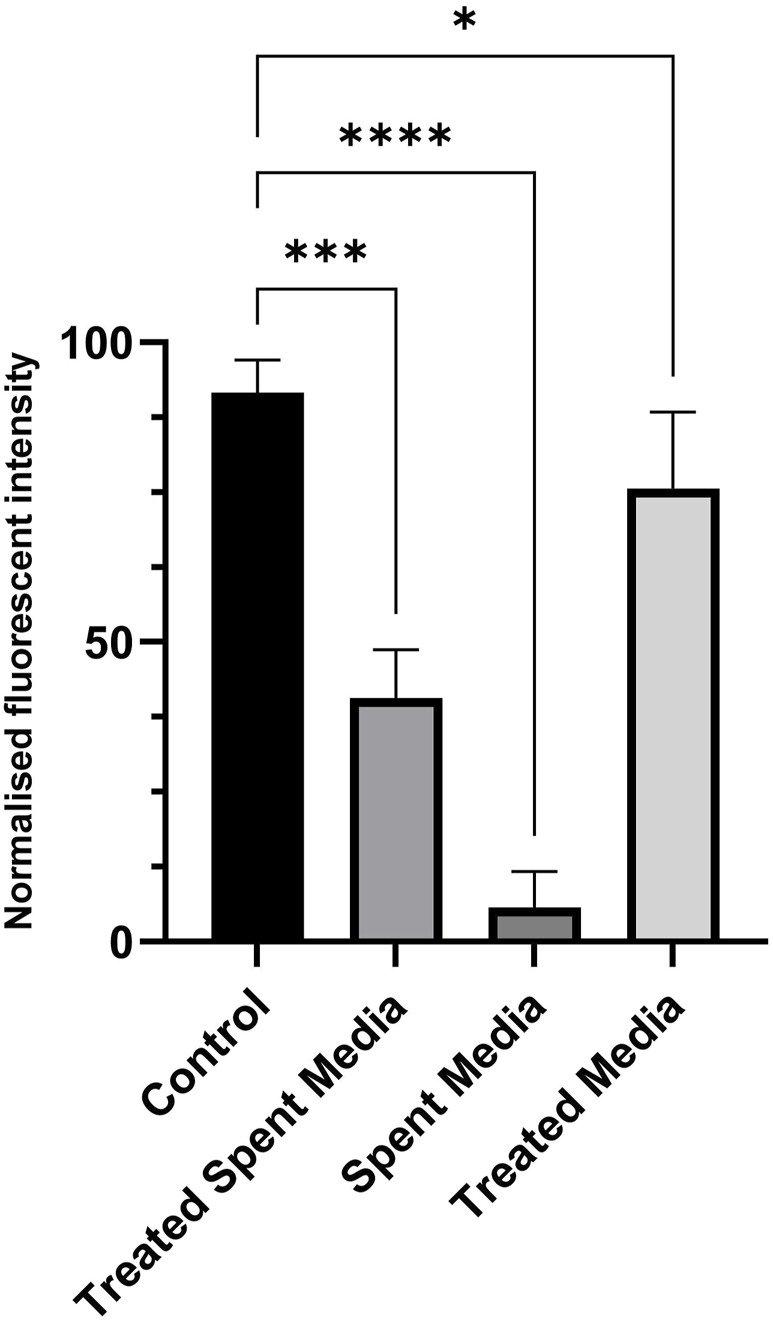
Short-term growth and proliferation of LSCs over 3 days with fresh media (control), treated spent media, spent media, and treated fresh media. *, **, ***, and **** indicate different significance levels. Statistical significance levels were evaluated using one-way ANOVA with Duncan’s multiple range test.

**FIGURE 5:**
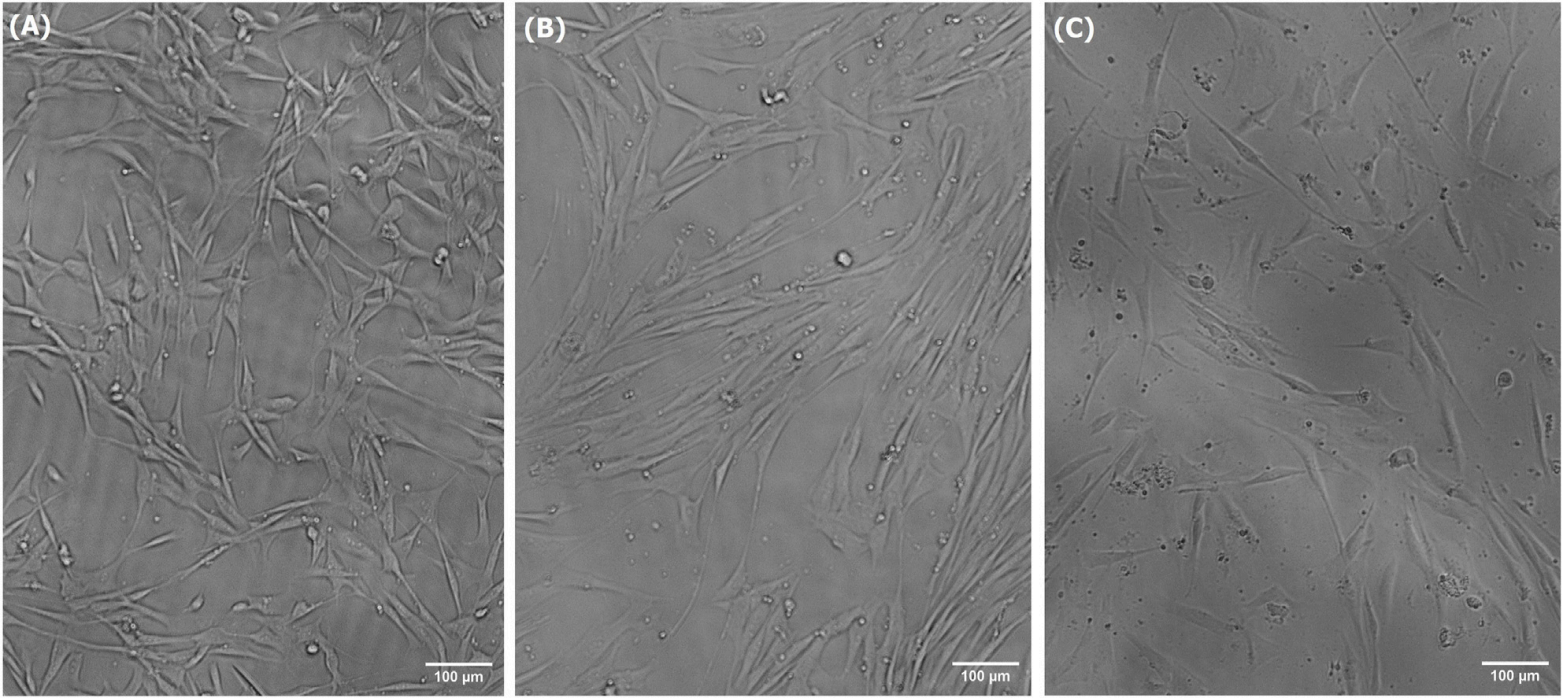
Bright-field images of LSCs grown for 3 days in fresh media **(A)**, treated spent media **(B)**, and spent media **(C)**.

**FIGURE 6:**
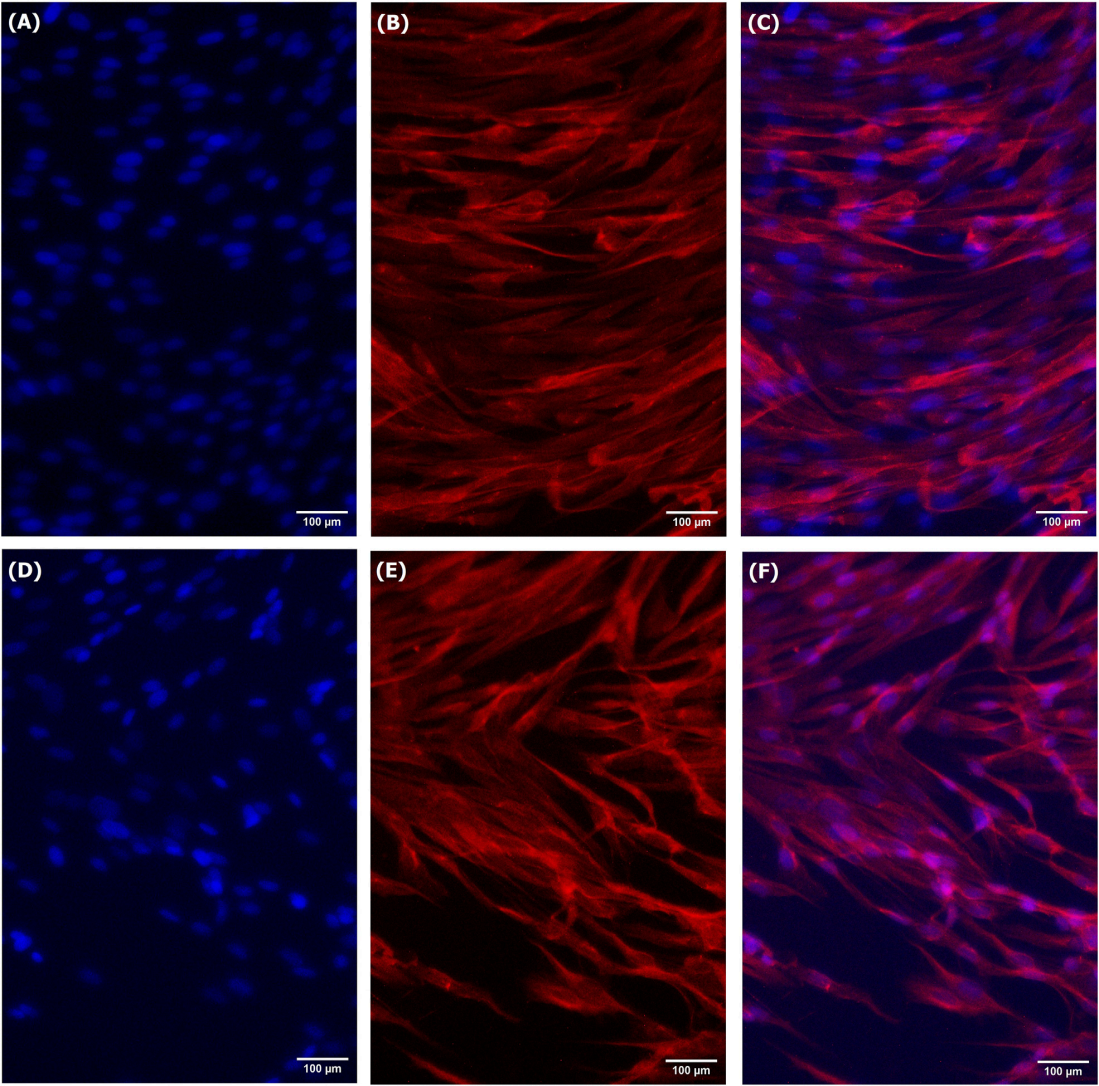
Immunofluorescent staining of LSCs grown for 3 days in treated spent media [**(A)** DAPI, **(B)** Pax7, and **(C)** Merge] and spent media [**(D)** DAPI, **(E)** Pax7, and **(F)** Merge].

Short-term growth, CyQUANT, and PAX7 immunostaining assays were used in this study to characterize the growth, activity, and viability of LSCs cultured in treated spent media. The spent media treated using the alkalization-stripping ammonia removal strategy optimized in this study supported significantly greater cell growth (*p = 0.0002*) than the untreated spent media (*p* < *0.0001*), as illustrated in Figure 4. However, treated media also significantly (*p* < 0.05) decreased cell growth, which can be ignored (Figure 4). Bright-field images and the morphology of LSCs in Figure 5 also showed that treated spent media were more capable of improving LSC growth than spent media in this study. Immunostaining and CyQUANT assays also confirmed the short-term growth evaluation (Figure 6). These results demonstrate a remarkably enhanced proliferation of LSCs grown in treated spent media compared to those grown in the untreated spent media. This finding suggests that the ammonia removal strategy optimized in this study can effectively transform spent media into a culture medium capable of substantially supporting the maintenance and growth of LSCs.

### 3.4 Recycling strategies

**FIGURE 7:**
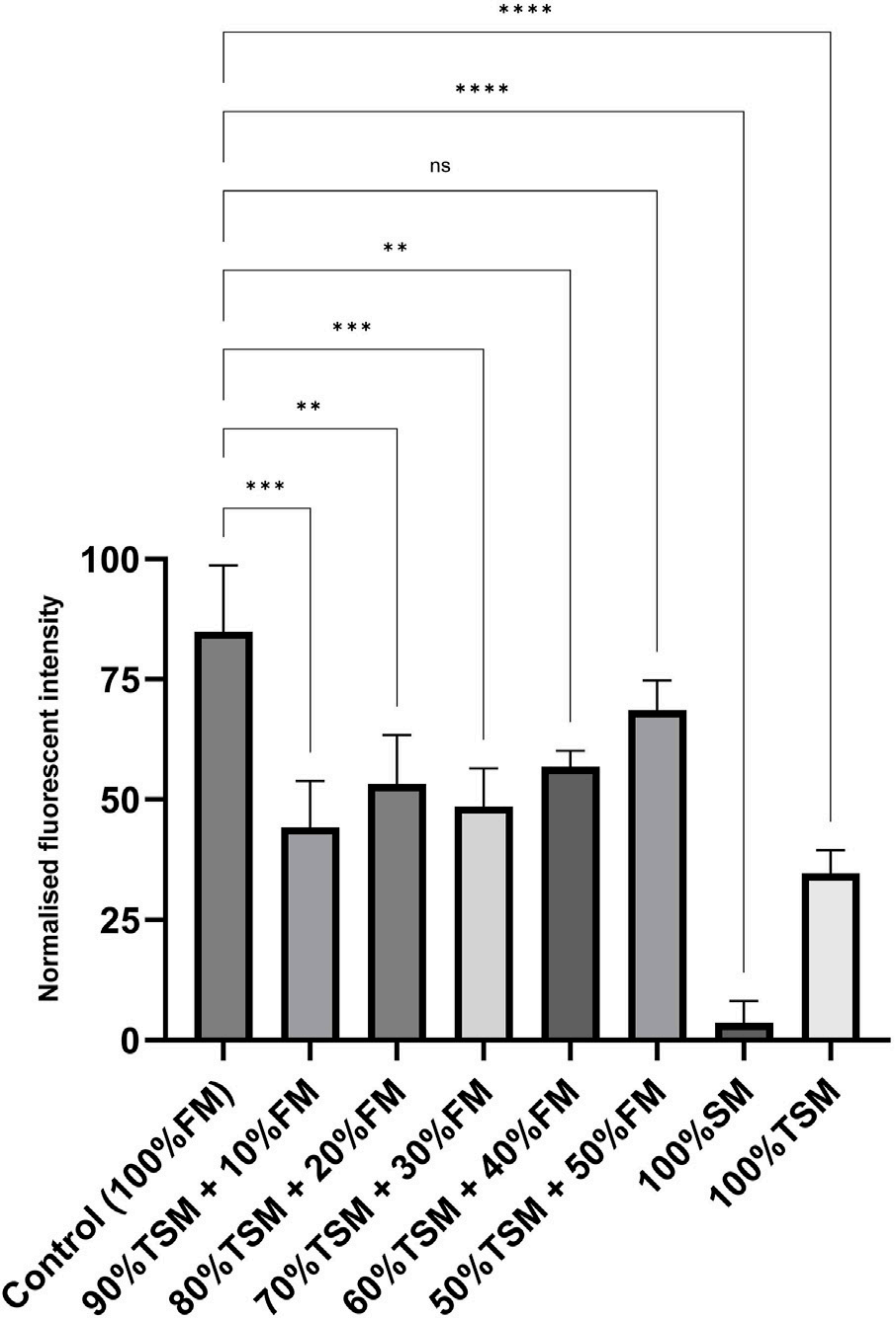
Short-term growth and proliferation of LSCs over 3 days with fresh media (control); 90:10 ratio of TSM to FM; 80:20 ratio of TSM to FM; 70:30 ratio of TSM to FM; 60:40 ratio of TSM to FM; 50:50 ratio of TSM to FM; and spent media and treated spent media. TSM, treated spent media; FM, fresh media; SM, spent media. *, **, ***, and 002A*** indicate different significance levels. Statistical significance levels were evaluated using one-way ANOVA with Duncan’s multiple range test.

We evaluated the short-term growth of LSCs in different formulations of treated spent media and fresh media, including ratios of 90:10, 80:20, 70:30, 60:40, and 50:50 (the treated spent media to fresh media), to assess and optimize recycling strategies. As shown in Figure 7, the recycling strategy utilizing a 50:50 ratio of treated spent media to fresh media significantly enhanced the growth rate of LSCs compared to the other formulation ratios. Furthermore, no significant difference was observed in the short-term growth rate of LSCs cultured under this recycling strategy compared to those cultured in fresh media.

## 4 Discussion

Mass biomanufacturing of a great diversity of cellular agricultural products, especially cultivated meats, has been considered one of the most promising and sustainable aspects of these novel technologies. On the other hand, scaling up and biomanufacturing in bioreactor systems are crucial components and the main technical keys to developing this innovative industry (Rischer et al., 2020; Eibl et al., 2021). Considering the long-term economic costs and the cost of the medium throughout the industrialization of this innovation, providing sustainable culture media by developing alternatives such as plant-based medium formulation and the spent media recycling strategies could be valuable and helpful in overcoming these challenges (O'Neill et al., 2021; Nikkhah et al., 2023). Because reusing the spent media is substantially more sustainable, cost-effective, and environmentally friendly compared to alternative culture medium formulations, we attempted to develop and optimize an affordable, rapid, user-friendly, and sustainable strategy for recycling the spent media in cultivated meat production. The most prominent challenge in media recycling is the inhibitory effects of the accumulation of metabolic by-products, mainly the secondary metabolites, against the growing cells in the medium culture (O'Neill et al., 2021; O’Neill et al., 2022; Ye et al., 2018).

Ammonia and lactate are the principal toxic metabolites with inhibitory effects on cell growth and activity, which should be removed from the spent media to achieve an efficient recycling strategy (O'Neill et al., 2021; Wynne et al., 2024). The lactic acid in the spent medium is produced through glycolysis and amino acid catabolism. At higher glucose concentrations, most of the glucose content is converted into lactic acid; however, at lower concentrations, most of it is oxidized to carbon dioxide. At concentrations above 40 mmol/L, lactic acid is more susceptible to limiting cell growth and yield by altering the pH and mainly affecting enzymatic activities, such as the inhibition of lactate dehydrogenase activity (Yang et al., 2023; O'Neill et al., 2021; O’Neill et al., 2022). Ammonia in spent media is mainly derived from the metabolism of some specific primary products such as L-glutamine. L-glutamine is essential for nucleic acid and protein synthesis in cells. It is highly unstable and immediately degraded and hydrolyzed to ammonia ions and L-glutamate or glutamic acid in culture media (Yang et al., 2023; O'Neill et al., 2021; Wynne et al., 2024). Ammonia ions can negatively and directly affect cellular metabolism by inhibiting glutamate dehydrogenase activity and ATP production through cytoplasmic acidification. A range of 1.8–33 mmol/L concentration of ammonia ions induces inhibitory and toxic effects on most mammalian cells; however, some cell types, such as Vero cells, are insensitive to ammonia (Saldanha et al., 2023; Hubalek et al., 2022). Since the growth and activity of the mammalian cells are believed to be more affected by the presence of ammonia ions in spent media, this study prioritized ammonia removal as a key focus in developing a strategy for spent media recovery.

In this study, we went through four practical wastewater treatment-based ammonia removal strategies. We found the alkalization-stripping strategy to be more efficient than other methods in removing ammonia ions significantly and preserving the remaining glucose contents in cell culture spent media. Glucose preservation was prioritized as a key indicator of nutrient retention due to its essential role in energy metabolism, and while high pH may cause denaturation or precipitation, immediate neutralization post-treatment helped mitigate such risks, as supported by the stable cell morphology and growth rates. While replenishing glucose post-treatment is a viable approach, preserving glucose offers a more integrative recycling strategy, maintaining medium integrity and reducing the need for re-supplementation of multiple components (Yang et al., 2025; Haraguchi et al., 2022; Thyden et al., 2025). All methods that were developed and used to remove ammonia ions during wastewater treatment, fermentation bioprocess, and microbial and cell media culture recovery were classified into five main strategies, namely, the modification of cell culture formulation, genetic engineering techniques, biocatalytic methods, adsorption, and electrochemical procedures, all of which have several challenges and potential issues (Yang et al., 2023; Wynne et al., 2024; Hubalek et al., 2022). One promising and effective technique to reducing the accumulation of toxic cellular secondary metabolites is cellular engineering, involving targeted amplification or disruption of some specific genes, such as glutamine synthetase and lactate dehydrogenase A genes, in mammalian cells to reduce ammonia and lactic acid production in culture media during cellular growth and activity. Since the cultured cells are ultimately intended for consumption and consumer concerns regarding genetically modified foods persist, such strategies are not advisable for use in cultivated meat production and related industries (Yip et al., 2014; Martinez-Turrillas et al., 2022). Some cultivation-based strategies have also been designed and employed to streamline the recycling process of cell culture media, including fed-batch cultivation and optimization of culture medium formulations to reduce ammonia production by using alternatives to L-glutamine, such as L-alanine and L-glutamine dipeptides. Due to their high cost and limited cost-effectiveness, cultivation-based methods have not been regularly recommended for ammonia removal during cell culture cycling. On the other hand, any modifications in culture medium formulations significantly affect the efficiency of mammalian cell growth (Ritacco et al., 2018; Yao and Asayama, 2017).

Physicochemical methods such as adsorption and precipitation strategies for removing ammonia nitrogen have emerged as popular procedures for wastewater treatment and have recently been used as more favored, cost-effective, user-friendly, and efficient approaches to remove the toxic compounds of mammalian cell metabolism (Yang et al., 2023; Wynne et al., 2024). The materials used for the precipitation or adsorption of toxic metabolites must satisfy key requirements, including strong preservation of essential nutrients, minimal impact on medium pH value, and non-cytotoxicity. Some of these materials that are routinely used as absorbents for wastewater treatment and recommended to be employed to remove accumulated toxic compounds for culture medium recycling are zeolite, TiO_2_ (TiO_2_-NPs), activated carbon, and zirconium phosphate (Yang et al., 2023; Elliott et al., 2020). Zeolites are crystalline aluminosilicates with a structure containing pores filled with water molecules and cations, permitting reversible dehydration and ion exchange (exchange with NH_4_^+^). The zeolite adsorption approach was previously applied as a promising strategy to remove ammonia from cell culture media; however, we found that it is not as efficient and rapid as the alkalization-stripping approach used in this study (Kameda et al., 2021; Ma et al., 2022). Due to the presence of different amino acids and some specific inorganic ions, such as Mg^+^, K^+^, Ca^+^, and Na^+^, in cell culture media, which can be competitively adsorbed, the efficiency of zeolites is significantly reduced for ammonia adsorption compared with the other strategies (Yang et al., 2023; Kameda et al., 2021). Alternatively, zirconium phosphate, resin, and activated carbon can also be used as substitutes for zeolites to remove ammonia from spent media as an adsorption approach; however, the presence of inorganic ions in media still remains a significant challenge (Yang et al., 2023; Wynne et al., 2024; Gil-San-Millan et al., 2021).

In this study, the adsorption of ammonia ions by TiO_2_-NPs and struvite precipitation approaches proved ineffective in removing NH_4_^+^ from spent media; however, these strategies were strongly able to remove ammonia within wastewater applications. These methods were not previously employed for the treatment of cell culture spent media and were only successfully used for wastewater treatment. Several researchers reported that TiO_2_-NPs are capable of photocatalytic degradation or photodegradation of ammonia ions in wastewater. The UV-activated TiO_2_ as a catalyst can photochemically react with ammonia ions and turn them into harmless nitrogen and hydrogen gases (Liu et al., 2021; Haruna et al., 2022). As previously reported by Shavisi et al. (2014), one of the most effective parameters for the removal efficiency of the treatment by TiO_2_ is the initial concentration of ammonia ions. They found that the maximum ammonia removal efficiency can be achieved when the initial concentration of the ammonia ions is 10 mmol/L. Since the initial concentration of ammonia in spent media is typically below 5 mmol/L, the results obtained in this study align with expectations and, therefore, were predictable (Shavisi et al., 2014). Yang et al. (2025) recently employed sodium zirconium phosphate for the adsorption and removal of ammonia ions from spent cell culture media in cultivated meat applications, reporting the method to be highly biocompatible and suitable for repeated use in media recycling (Yang et al., 2025).

Some other ammonia separation approaches, such as the precipitation method with struvite formation, were also used to remove and recover nitrogen and phosphorus from the wastewater. Struvite or magnesium ammonium phosphate hexahydrate (MgNH_4_PO_4_.6H_2_O) forms when ammonium (NH_4_^+^), magnesium (Mg^2+^), and phosphate (PO_4_^3−^) ions are present in sufficient concentrations. According to the mechanism of struvite formation, the presence of ammonia facilitates the precipitation of ions through the addition of extra amounts of phosphate and magnesium ions, leading to the formation of struvite crystals (Lorick et al., 2020; Wu and Vaneeckhaute, 2022). Due to the significantly higher amount of ammonia and phosphate ions in wastewater, struvite precipitation was observed as an efficient and cost-effective strategy for the recovery of these compounds in wastewater treatment (Wu and Vaneeckhaute, 2022). However, significantly lower concentrations of ammonia ions in spent media present a substantial challenge in employing this practical strategy for ammonia removal from spent media as we also observed no significant change in the ammonia content after struvite precipitation in this study (Yang et al., 2023). As previously reported by Simoes et al. (2018) and Jing et al. (2019), alkaline pH (pH = 9.3–10) mediates ammonia removal through struvite precipitation. On the other hand, ammonia ions can be efficiently removed in alkaline pH values using the air-stripping process, which is considerably more cost-effective and environmentally friendly than the struvite precipitation strategy; therefore, we were encouraged to develop and evaluate the fourth approach, the alkalization-stripping strategy (Simoes et al., 2018; Jing et al., 2019).

Among all the approaches developed and evaluated in this study, we found the alkalization-stripping strategy to be the most efficient, rapid, cost-effective, and environmentally friendly method to recover ammonia from the spent media. As discussed before, due to the limited amounts of phosphate ions and low concentrations of ammonia ions in spent media, the alkalization-stripping method can be more technically efficient and economically feasible for removing ammonia from spent media than struvite precipitation; however, both approaches have been considered highly efficient for ammonia removal and nutrient recovery during wastewater treatment, as comprehensively reviewed and discussed by Wynne et al. (2024) and Wu and Vaneeckhaute (2022). The alkalization-stripping method, but at higher temperatures (more than 100°C), has also been previously used for ammonia recovery from cow manure and landfill leachate. Since there are some specific compounds in the spent media that are highly sensitive to thermal processing, mainly proteins, we implemented the procedure at room temperature. Ammonia nitrogen can exist in two forms in aqueous solutions: the ionic form (NH_4_^+^) or dissociated ammonia and the undissociated form of ammonia (NH_3_, free or volatile forms of ammonia) or ammonia gas. There is a balance between ionic ammonia and ammonia gas according to the pH value of the solution. Alkalinity or pH values higher than 7 contribute to the formation of ammonia gas (from the ionic form of ammonia in the aqueous solution), which is volatile and can immediately be separated from the aqueous solution (Kinidi et al., 2018; Lorick et al., 2020; Wu and Vaneeckhaute, 2022).

Performing a stripping process following the alkalization of the solution could effectively be a practical strategy to recover the ammonia ions from the aqueous solution in the form of gaseous or volatile ammonia. At a pH value of 9.25, 50% of ammonia ions volatilized immediately to free ammonia. As the pH of the aqueous solution further increases to 11, the proportion of free ammonia increases significantly, reaching up to 99%. More than 50% of the volatilized ammonia ions are immediately removed from the solution in the gaseous form, and the remaining ions can be separated gradually throughout the stripping process (Lorick et al., 2020; Wu and Vaneeckhaute, 2022). Since most of the mammalian cells used in cellular agriculture for cultivated meat production can only grow within neutral pH ranges, the treated spent media subjected to the alkalization-stripping approach must be neutralized and adjusted back to the original pH value of the cell culture medium (pH = 7.42) (Leese et al., 2021; Synoground et al., 2021). Ultimately, we designed an appropriately modified approach for ammonia removal from cell culture spent media that can subsequently be subjected to optimization for enhanced efficiency and effectiveness.

RSM-CCD is a convenient and practical method that can be widely used in different disciplines for optimization and modeling of the process parameters and their interactions (Chelladurai et al., 2021). In the present study, we successfully modeled and optimized the process parameters of the alkalization-stripping approach, including the pH value and process duration, for ammonia recovery from spent media using the RSM-CCD. The analysis of variance and statistical parameters demonstrated that a quadratic model represented by a second-order polynomial equation was significantly fitted to describe and optimize the process parameters. The optimal process parameters for ammonia removal from spent media were ultimately determined to be a pH of 12 followed by a 15-min stripping step. We also observed that ARE (the response) increases with increasing pH values while remaining independent of the duration of the process. As previously discussed in this study, these results can be attributed to the formation of volatile or free ammonia from the ammonia ions, along with the immediate separation of over 50% of the ammonia gas from the spent media following the alkalization step (Kinidi et al., 2018; Lorick et al., 2020; Wu and Vaneeckhaute, 2022).

We were finally successful in removing more than 82% of the ammonia ions from the spent media using the optimized alkalization-stripping method in this study. There are a limited number of studies making efforts to remove ammonia from the cell or microbial culture spent media. Kameda et al. (2021) evaluated zeolite and Prussian blue to remove ammonia from culture solutions. They reported that zeolite and Prussian blue were able to recover the ammonia from the aqueous solutions with maximum efficiencies of 60% and 10%, respectively. Wu and Vaneeckhaute (2022) also tried to develop a clinoptilolite-based platform for ammonia removal from the cell culture spent media, and they were finally able to remove 35.8% of the ammonia ions from the culture solution. To develop the final optimized strategy for ammonia recovery from spent media, we conducted cell growth characterization assays on LSCs to evaluate the effectiveness of the treated spent media.

Our findings demonstrated that treated spent media significantly enhanced the proliferation of LSCs without any considerable cellular morphological changes compared to those grown in spent media. Due to the direct toxic effects of ammonia on cellular metabolism and energy transfer, higher levels of ammonia recovery from or reduced accumulation of ammonia ions in cell culture media contribute to enhancing cell proliferation and growth efficiency (Yang et al., 2023; Wynne et al., 2024; Yao and Asayama, 2017; Pasitka et al., 2024). We also evaluated different recycling strategies and found that the 50:50 ratio of treated spent media to fresh media can enhance the growth rate of LSCs cultured in this formulation without any significant difference compared to the growth rate of LSCs cultured in fresh media. The treatment of spent media using the approach developed and optimized in this study represents a sustainable strategy with significantly reduced water and energy consumption and lower CO_2_ emissions. This optimized recycling method offers a cost-effective and environmentally friendly solution for ammonia removal in the recycling of spent media, contributing to the sustainability of cultivated meat production (Yang et al., 2023; O'Neill et al., 2021; Barbaroux et al., 2024). We strongly recommend that future studies investigate not only the effects of this ammonia removal treatment on the growth rates of other cell types and nutrients beyond glucose in cell culture media but also explore multi-cycle reuse and dynamic recycling formulations as promising strategies to further enhance the sustainability and efficiency of the process.

## 5 Conclusion

In conclusion, ammonia is a toxic compound for mammalian cells and should be removed from cell culture spent media to enable the development of a media recycling strategy in cultivated meat production. We evaluated four practical wastewater treatment-based ammonia removal strategies to recover ammonia from cell culture spent media and found the alkalization-stripping approach to be more efficient and rapid than other strategies for removing ammonia ions while preserving the remaining glucose content. The selected approach was successfully modeled and optimized using RSM-CCD, and the optimal process parameters were determined to be a pH of 12 followed by a 15-min stripping process, achieving more than 82% ARE. Compared to spent media, treated spent media improved the growth rate of LSCs without any significant morphological change. Ultimately, a 50:50 formulation of treated spent media and fresh media was suggested as an efficient, cost-effective, and environmentally friendly recycling approach, offering a practical solution to support sustainable media cycling in cultivated meat production.

## Data Availability

The original contributions presented in the study are included in the article/supplementary material; further inquiries can be directed to the corresponding author.
